# Language and communication skills in multilingual children on the autism spectrum: A systematic review

**DOI:** 10.1177/13623613221147780

**Published:** 2023-01-11

**Authors:** Christina Sophia Gilhuber, Tracy Jane Raulston, Kasie Galley

**Affiliations:** 1 The Pennsylvania State University, USA; 2 Action Behavior Centers, USA

**Keywords:** autism, bilingualism, communication and language, multilingualism

## Abstract

More than half the global population is estimated to be multilingual, yet research on autistic children who grow up in multilingual environments remains scant. We conducted a systematic review of peer-reviewed studies on multilingualism in autistic children and its impact on children’s language and communication skills. Following Preferred Reporting Items for Systematic Reviews and Meta-Analyses guidelines, our search identified 22 group comparison studies published from 2011 to 2022. The current review addressed two main questions: (a) Which language and communication skills of multilingual autistic children were assessed and how; and (b) how the skills of multilingual autistic children compared with their peers. Semantic-related skills were most frequently reported, while phonology and pragmatics were the most underreported dimensions of language. Most reviewed studies used a combination of direct and indirect language assessments. Available research provides no indication that being exposed to more than one language has adverse effects on the communication skills of autistic children. Although multilingual autistic children often have common autism characteristics, such as pragmatic difficulties affecting their communication skills, preliminary findings also indicate that they may share some advantages of multilingualism with their multilingual nonautistic peers. Studies often excluded participants with co-occurring diagnoses, which may have impacted the generalizability of the findings.

**Lay Abstract:**

Both parents and service providers have voiced concerns about the potential negative impact of exposure to multiple languages on the language and communication skills of autistic children. The current literature review summarized research that assessed the language and communication skills of multilingual autistic children in comparison with their autistic and nonautistic peers. After a comprehensive search, 22 relevant publications were identified that met the inclusion criteria of the current review. Thirteen studies used both direct (directly administered screening/diagnostic tools) and indirect language assessments (e.g. parent questionnaires). Receptive and expressive vocabulary was the most frequently assessed language skill. Available research does not support the assumption that bilingualism has negative effects on the language and communication skills of autistic children. The language and communication skills of multilingual autistic children frequently resembled their monolingual autistic peers in both strengths and areas of growth. Preliminary findings indicate that multilingual autistic children may share some advantages of multilingualism with their multilingual nonautistic peers. Studies often excluded participants with intellectual disabilities or complex communication needs, which means that a large population of autistic children is not yet represented in research about the effects of multilingualism.

Globally, half of the population is estimated to be bilingual (Grosjean, 2010). One-fifth of the American population and more than one-third of the Canadian population is bilingual (Grosjean, 2013). With even higher bilingual rates for Africa, Asia (Grosjean, 2013), and Europe (European Commission, 2012), millions of children are educated in a language other than, or in addition to, their first language (Grosjean, 2010). The high numbers of bilinguals and children being raised in multilingual environments allow the assumption that a significant proportion of children on the autism spectrum are exposed to more than one language. For example, Trelles and Castro (2019) estimated that up to 25% of children on the autism spectrum grow up in bilingual environments.

Language skills in children on the autism spectrum encompass a spectrum of unique abilities ranging from complex communication needs to typical development (Hudry et al., 2010). For this reason, both parents and professionals have voiced concerns about the effects of bilingual exposure on the language development of children on the autism spectrum (e.g. Kremer-Sadlik, 2005). The available research on the language and communication skills of autistic children shows that bilingualism appears to have no adverse effects on children’s language and communication skills (e.g. Yu, 2016). The current review aims to synthesize (a) which dimensions of language (phonology, morphology, semantics, syntax, pragmatics) have been investigated so far and how the language and communication skills of multilingual children on the autism spectrum have been assessed, and (b) how the language and communication skills of multilingual children on the autism spectrum compared with their peers. Specifically, we examined the extent to which the language skills of multilingual children on the autism spectrum resemble those of their monolingual peers on the autism spectrum and to what extent they resemble the skills of their nonautistic multilingual peers. In the current review, we identified 22 group comparison studies that were published prior to January 2022.

## Multilingualism

Definitions of multilingualism and bilingualism vary (Cenoz, 2013). Bilingualism is the use of multiple languages or dialects in daily life (Grosjean, 2013; Petersen et al., 2012). In addition, bilingualism is defined based on different factors, including proficiency and exposure (Surrain & Luk, 2017). Bilingual exposure varies greatly, including differences in age and amount of exposure (Luk & Bialystok, 2013). Regarding the age of exposure, researchers generally distinguish between simultaneous and sequential bilinguals (Paradis et al., 2021). Simultaneous bilinguals are exposed to two languages during their infant and toddler years, while children exposed to a second language after their third birthday are typically referred to as sequential bilinguals (Paradis et al., 2021).

The regular use of more than two languages is generally described as multilingualism (e.g. European Commission, 2007). In the current review, the term multilingualism will be used to include individuals who speak two languages as well as those who speak more than two languages.

## Language dimensions and development

Language encompasses spoken, written, and nonverbal communication and includes five dimensions: phonology, morphology, semantics, syntax, and pragmatics (Kortmann, 2005). The dimension of phonology (including phonetics) is concerned with the sounds of a language and their production, perception, and function (Skandera & Burleigh, 2016). The dimensions of language also include morphology (i.e. meanings of internal structures of words), semantics (i.e. meanings of words, phrases, and sentences), and syntax (i.e. principles that govern the construction of phrases and sentences; Kortmann, 2005; Skandera & Burleigh, 2016). The dimension of pragmatics involves how individuals utilize and adapt language within social and cultural contexts (Bornstein et al., 2014; Gleason, 2017). Social pragmatic development extends beyond spoken language and includes nonverbal and preverbal skills such as eye contact (Carbone et al., 2013), communicative gestures (Franchini et al., 2018; Smith et al., 2018), turn-taking (Edmister & Wegner, 2015), and joint engagement (Kasari et al., 2006).

The first few years of a child’s life contain significant language developmental milestones. Nonverbal communication and communicative intent begin to develop before the first words are typically voiced around 12 months (Tager-Flusberg et al., 2005). Around 18–20 months, children usually start combining words to form two-word phrases (Fenson et al., 1994). Semantic and syntactic development consistently progresses further in the following years (Tager-Flusberg et al., 2005).

## Language and communication skills in children on the autism spectrum

While language skills are no longer part of an autism diagnosis, according to the fifth edition of the *Diagnostic and Statistical Manual of Mental Disorders* (*DSM*; American Psychiatric Association [APA], 2013), autistic individuals show high heterogeneity in their language profiles (e.g. Tager-Flusberg, 2006). Children on the autism spectrum display a wide range of verbal and nonverbal skills (Noens & van Berckelaer-Onnes, 2005), including significant language delays (Weismer et al., 2010), language regression (Lord et al., 2004), and deficits in social pragmatic skills such as joint attention (Warreyn et al., 2005) and figurative speech (Baird & Norbury, 2016). The language development of children on the autism spectrum can present with difficulties in both receptive and expressive language skills (Hudry et al., 2010). Hudry and colleagues found that (a) children on the autism spectrum performed below age norms, and (b) the development of receptive language skills was generally more delayed than expressive language skills.

In addition, speech development varies significantly among autistic children and has been found to be both delayed and divergent from common milestones (Gerenser & Lopez, 2017). Prevalence estimates indicate that approximately 30% of individuals on the autism spectrum do not acquire functional phrase speech (Anderson et al., 2007; Wodka et al., 2013).

While autistic children have been found to score lower on language and communication assessments than nonautistic controls on a group level, language profiles are highly heterogeneous (e.g. Tager-Flusberg, 2006). Therefore, language and communication skills across different domains should be assessed not only for monolingual children but also for multilingual children on the autism spectrum.

## Multilingual language development

Lexical development generally happens at a similar pace for monolingual and bilingual children (Genesee, 2003; Petitto et al., 2001). Early developmental milestones like babbling and first words emerge at a similar timeline for simultaneous bilingual children and monolingual children in at least the bilingual children’s dominant language (Paradis et al., 2021). The timeline for sequential bilingual children’s non-dominant language development may differ (Paradis et al., 2021). This connects to the fact that language development is dependent on both quality and quantity of language input (Paradis et al., 2021).

The effect of bilingualism on phonetic processing and phonological acquisition depends on the conformities of the linguistic profiles of the two languages (Havy et al., 2016). In addition to the phonological level, cross-linguistic transfer across the languages of multilingual speakers also occurs for the language dimensions morphology, semantic, and syntax (McLeod et al., 2017).

Simultaneous bilinguals develop their languages neither in perfect synchrony nor in isolation (Paradis et al., 2021). The interdependence of the development of both languages of simultaneous bilinguals may be why the overall language development of this population is not significantly delayed compared with their monolingual peers (Paradis et al., 2021). In 1989, Grosjean argued that a bilingual is not equal to two monolinguals in one mind. Research has since found that both languages of bilinguals are constantly activated parallelly, even when activation of only one language is required (e.g. Van Assche et al., 2009). These cross-language interactions have also been found to be bidirectional (Kroll et al., 2015), meaning that not only does the first language influence the second language, but vice versa is also true in proficient bilinguals (Dussias & Sagarra, 2007; Van Hell & Dijkstra, 2002). It is hypothesized that controlling the constant competition between two languages may lead to bilinguals performing better on executive functioning tasks (Kroll et al., 2012).

Another common occurrence in bilinguals’ communication patterns is what is known as code-switching or code-mixing (Paradis et al., 2021), which is the alternating use of two languages within the same conversation or even the same utterance (Genesee, 2003; Kaushanskaya & Crespo, 2019; Wei, 2000). Code-switching is a natural occurrence in bilingual settings, not interference between languages (Kroll et al., 2012). Available evidence also indicates that syntactic rules of different languages, such as word order, are rarely confused by bilingual children (Beauchamp & MacLeod, 2017).

Language development for sequential bilinguals is more individualized than the language development of simultaneous bilinguals and is influenced by various factors (Paradis et al., 2021). Internal factors, such as age of acquisition and personality, and external factors, such as amount and quality of second language exposure, influence second language development (Paradis et al., 2021).

In general, both quality and quantity of language input have been found to predict language acquisition in bilingual children (Paradis, 2018). Language environments, therefore, play an important role in bilingual language development (Paradis, 2018).

The American Speech-Language-Hearing Association’s (ASHA; 2004) guidelines for the assessment of bilingual children by speech-language pathologists state that in addition to language use, language proficiency should be measured in each language. Two-language approaches have been found to provide a more accurate reflection of bilingual speakers’ proficiency than single-language assessments (e.g. Peña et al., 2016). Core et al. (2013) have also criticized single-language comparisons as inaccurate reflections of the true language skills of bilingual children and have suggested the use of total vocabulary scores (the sum of words known across both languages) as opposed to conceptual vocabulary scores, wherein the concept of a word counts representatively for both languages or single-language comparisons. For this reason, in the current review, we coded the included publications for the languages that were assessed as well as the type of assessment.

## Multilingualism in children on the autism spectrum

Although increasing, research on bilingualism in children on the autism spectrum remains scant to date. In addition to the group comparison studies that have been the focus of previous reviews (e.g. Drysdale et al., 2015; Lund et al., 2017), there are single-case studies investigating different aspects of bilingualism in autistic children (e.g. Aguilar et al., 2016; Seung et al., 2006; Yu, 2016). In a single-case study of a 5-year-old bilingual boy on the autism spectrum, Yu (2016) found that a child strategically used code-switching to switch between Mandarin and English depending on the demands of context as well as personal preference. Another single-case study found that a 6-year-old Spanish–English bilingual on the autism spectrum preferred to receive instruction in Spanish, which was their home language (Aguilar et al., 2016). For a comprehensive review, see Yu (2018).

Research investigating the effects of monolingual and bilingual interventions for multilingual children on the autism spectrum (e.g. Lang et al., 2011; Summers et al., 2017) is scant. Lang et al. (2011) compared the effects of providing intervention in both languages of a bilingual child on the autism spectrum and reported more positive effects on response accuracy and behavior when the intervention was provided in the home language. Summers et al. (2017) compared a monolingual and a bilingual intervention in an alternating treatment design for two participants and concluded that both provided similar benefits.

A few studies have interviewed parents of autistic children who were raised in multilingual environments (e.g. Howard et al., 2021; Ijalba, 2016; Yu, 2013). Parents of multilingual children on the autism spectrum have reported that professionals often advised them to speak only one language with their child (e.g. Fernandez y Garcia et al., 2012; Kremer-Sadlik, 2005), despite the fact that there is no scientific evidence to support the clinical recommendation that a monolingual environment is beneficial for the language development of children on the autism spectrum. On the contrary, advising parents to abandon one of their languages during interactions with their child has been found to have potentially negative effects on family interactions, such as parents feeling uncomfortable speaking a non-native language with their child (Fernandez y Garcia et al., 2012); children being excluded from family interactions (Kremer-Sadlik, 2005); and interactions being limited with monolingual family members (Jegatheesan, 2011).

Recent studies have reported on the perceptions of multilinguals on the autism spectrum regarding their own experiences. In their study on language profiles and social experiences of autistic adults, Digard et al. (2020) found that 33% of participants identified as bilinguals, and 37% reported knowing at least three languages. Participant responses indicated a positive association between bilingualism and social life quality (Digard et al., 2020). On a related study, Nolte et al. (2021) conducted a qualitative analysis of the survey responses of multilingual autistic adults and concluded a wide range of diverse language experiences among the participants. Participants reported various reasons for learning languages and listed a number of perceived benefits of being multilingual (Nolte et al., 2021). Howard et al. (2019) conducted semi-structured interviews with 11 bilingual children and adolescents on the autism spectrum between the ages of 7 and 14. The analysis of the interviews concluded that language environments have a significant influence on the individual’s perspective of their multilingualism. Specifically, those who were educated in multilingual settings reflected more positively on their multilingualism than their peers who were educated in monolingual contexts (Howard et al., 2019).

Building on previous reviews (e.g. Drysdale et al., 2015), the current review also focuses on group comparison studies that investigated how the language and communication skills of multilingual children on the autism spectrum compared with their peers. Comparison groups include monolingual autistic children, multilingual nonautistic children, and monolingual non–autistic children. Previous literature reviews on this topic have concluded that existing research does not support the concern that bilingual exposure might have any detrimental effects on the language and communication skills of autistic children (e.g. Conner et al., 2020; Drysdale et al., 2015; Garrido et al., 2021). The current review intends to expand on these findings by investigating to what extent the language and communication skills of multilingual autistic children resemble or differ from the skills of both their autistic and nonautistic peers. In addition, we synthesized the findings to highlight which aspects of language and communication have been assessed and how. There has been an increase in studies on the topic of multilingualism in autistic children in the past 5 years. Therefore, it is our aim to provide an updated synthesis of group comparison studies between multilingual children on the autism spectrum and their peers. Aiming to extend previous reviews, we intend to highlight which aspects of language have been assessed and how the language and communication skills of participants have been evaluated.

## Purpose of the present study

The purpose of this review was to identify and synthesize peer-reviewed publications on multilingualism in children on the autism spectrum. We sought to answer the following research questions:

1. What dimensions of language have been included in studies of multilingualism in autistic children, and how have they been measured?
2. How do the language and communication skills of multilingual autistic children compare with multilingual nonautistic children and monolingual autistic children?
2.1 To what extent do the language skills of multilingual autistic children resemble the language skills of multilingual nonautistic children?
2.2 Are commonly observed language features of autistic children observed to the same extent in multilingual autistic children as in monolingual autistic children?

## Method

### Protocol and eligibility criteria

A systematic literature review was conducted according to the guidelines of the Preferred Reporting Items for Systematic Reviews and Meta-Analyses (PRISMA; Moher et al., 2009). To be included in this review, studies had to (a) be published in English and in a peer-reviewed journal; (b) be of a quantitative design; (c) include multilingual autistic children between the ages of 1 and 12 years; (d) include at least one comparison group (i.e. monolingual autistic children; multilingual nonautistic children); (e) incorporate at least one language measure. Specifically, multilingual children were defined as those who were (a) proficient in two or more languages, (b) exposed to at least two languages regularly, or (c) exposed to each language for at least 20% of their lifetime.

### Search

We searched the databases ProQuest (ERIC), EBSCO (Academic Search Complete, PsycINFO), and Medline (PubMed). We included all records that were published prior to 8 January 2022, and met the eligibility criteria of this study. The lower bound limit for the publication date was 2011.

We conducted the database search choosing to focus on (a) children on the autism spectrum who (b) spoke or were exposed to more than one language. We employed an advanced search method that included various search terms for both categories. The following search terms were included in the first line: (autis* OR asperger* OR *ASD* OR *PDD-NOS* OR “pervasive develop*”). To identify multilingual participants, the following search terms were included in the second line: (biling* OR multiling* OR “*dual language*” OR “*second language*” OR “*heritage language*” OR “*English language learner*” OR “*limited proficiency*” OR *ESL* OR *ELL*). We used the AND feature to combine the two lines. For some search terms, truncations were used to include different variations of the term. We used database filters to limit the results to peer-reviewed publications written in English. The first and the third author independently conducted the search for each database. Agreement for search results was 100% for all databases. An ancestry search resulted in the identification of five additional articles. All five articles met all eligibility criteria and were included in the review.

### Study selection

**Figure 1.**
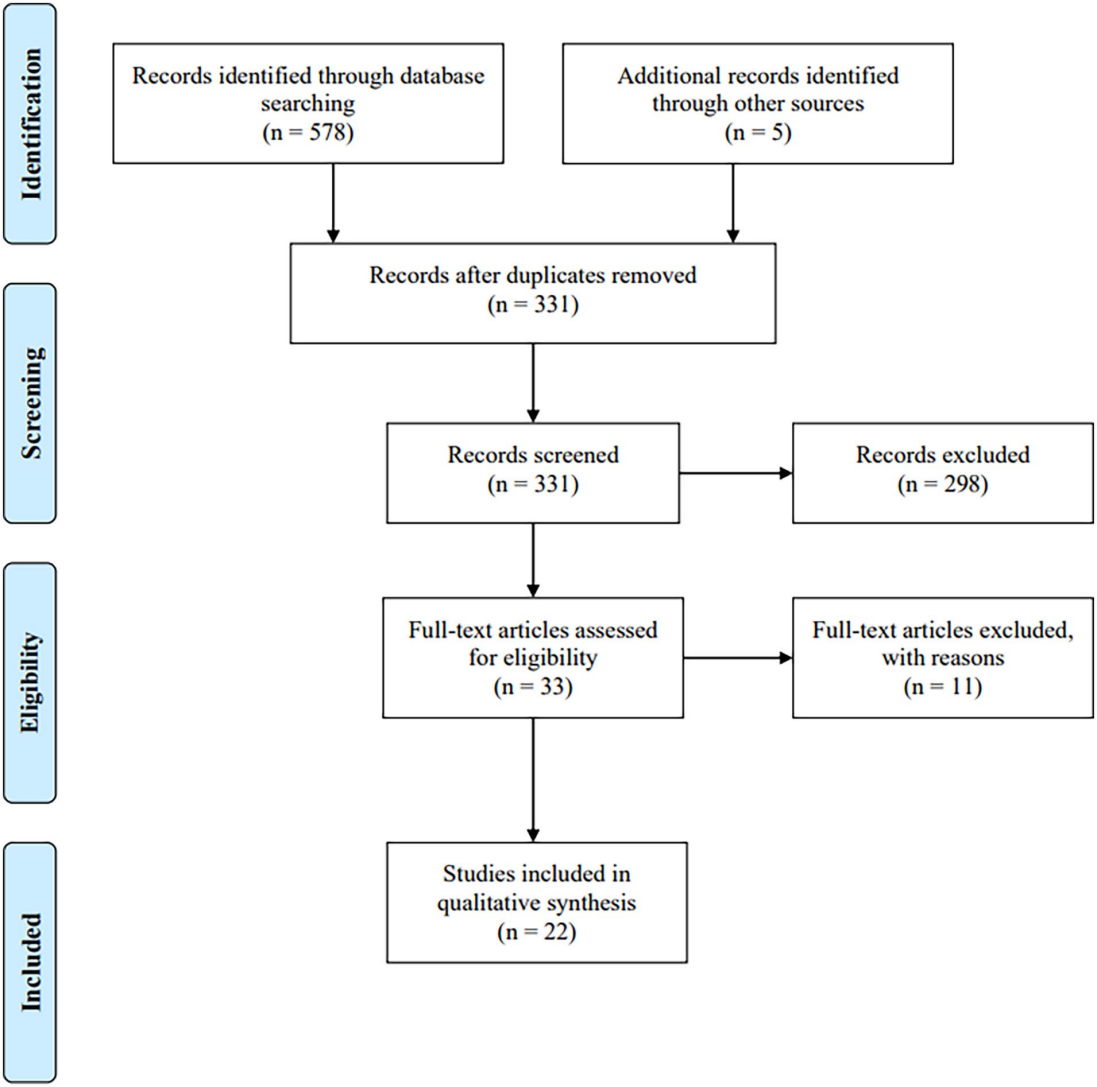
Flow diagram displaying the identification and selection of articles. *Note*. This figure is minorly adapted from the PRISMA flow diagram (Moher et al., 2009).

The search resulted in the identification of 578 publications. Adding in five articles that were identified through lineage search, we identified a total of 583 records. We excluded 252 duplicates and then screened the remaining 331 publications’ titles. For the records where eligibility could not be determined based on the title, we read the abstract. Thirty-three articles required a review of the complete text to assess eligibility. To ensure the reliability of the eligibility criteria, the first and the third authors independently reviewed the full text of the 33 articles. The inclusion decisions were in 97% agreement between the first and third authors. Any disagreements were solved through discussion and consultation with the second author. Twenty-two peer-reviewed articles met all eligibility criteria and were included in the current review (Figure 1).

### Data extraction and coding procedures

The first author coded all 22 articles identified in the current review. The coding forms included (a) study characteristics; (b) participant characteristics; (c) quality of evidence; (d) language measures (e.g. formal assessment); (e) language dimensions (e.g. phonology, pragmatics); and (f) study outcomes. Studies were coded for study identification criteria (i.e. authors; year of publication; country in which the study was conducted); study design (e.g. group matching criteria); and participant eligibility criteria (e.g., exclusion of participants with a co-occurring intellectual disability or complex communication needs). Participant demographics were coded for age, gender ratio, age at diagnosis, race/ethnicity, nonverbal IQ (NVIQ), languages spoken, time of bilingual language exposure (sequential vs simultaneous), and occurrence of language regression. Language measures were coded for the type of language measurement (direct vs indirect) and the language measure itself (e.g. Peabody Picture Vocabulary Test; Dunn & Dunn, 2007), and for which language (first or second language) scores were reported. Study outcomes were coded for statistically significant differences in the language and communication skills between bilingual children on the autism spectrum and their peers. To ensure inter-rater reliability for the coding process, the third author was trained in the coding process and independently coded 32% (*n* = 7) of the articles included in the current review. Articles were randomly selected for inter-rater reliability coding. Once the first and third authors completed the independent coding, all codes were compared. Overall agreement for the 42 coded items was 97% and ranged from 86% to 100%. Any disagreements were resolved through discussion.

### Community involvement

Community members were not involved in this study.

## Results

The current review synthesized 22 quantitative studies with publication dates ranging from 2011 to 2021 (Beauchamp et al., 2020; Gonzalez-Barrero & Nadig, 2017, 2019a, 2019b; Hambly & Fombonne, 2012; Hoang et al., 2018; Li et al., 2017; Meir & Novogrodsky, 2019, 2020, 2021; Ohashi et al., 2012; Peristeri et al., 2020; Petersen et al., 2012; Reetzke et al., 2015; Sen & Geetha, 2011; Sendhilnathan & Chengappa, 2020a, 2020b; Siyambalapitiya et al., 2022; Valicenti-McDermott et al., 2013, 2019; Vanegas, 2019; Zhou et al., 2019). All 22 publications included at least one language measure (e.g. Peabody Picture Vocabulary Test; Dunn & Dunn, 2007) and a minimum of one comparison group (e.g. monolingual children on the autism spectrum).

### Study characteristics

The great majority of studies (*n* = 19) used nonexperimental, descriptive research designs in which the researchers did not manipulate any variables (Mertler, 2021). Only the studies by Sendhilnathan and Chengappa (2020a, 2020b) and Siyambalapitiya et al. (2022) included an intervention. A majority of the studies (*n* = 20) were cross-sectional group comparison studies (e.g. Beauchamp et al., 2020; Ohashi et al., 2012; Petersen et al., 2012; Vanegas, 2019). Zhou et al. (2019) and Siyambalapitiya et al. (2022) were the only longitudinal studies included in the current review.

Fifteen studies administered formal assessments and tasks to evaluate participants’ language and communication skills (e.g. Gonzalez-Barrero & Nadig, 2017; Li et al., 2017; Petersen et al., 2012). Other studies retrospectively analyzed data from medical records (Vanegas, 2019) or multidisciplinary evaluations (Valicenti-McDermott et al., 2013, 2019).

Twelve of the studies were conducted in North America (e.g. Valicenti-McDermott et al., 2019; Vanegas, 2019). Four studies were conducted, at least in part, in Asian countries. In addition, one study occurred in Australia (Siyambalapitiya et al., 2022), one in Greece (Peristeri et al., 2020), and all participants in the studies by Meir and Novogrodsky (2019, 2020, 2021) lived in Israel.

### Participant characteristics

Participants’ ages ranged from 1 to 12 years. A total of 82% (*n* = 18) of the recruited participants were 10 years or younger (see Table 1). Gonzalez-Barrero and Nadig (2019a), Peristeri et al. (2020), and Vanegas (2019) included participants up to 12 years of age. Most of the participants were male. Participants spoke a variety of languages, with English, Spanish, and French being the most common languages.

**Table 1.** Study characteristics.

Publication	N	Gender ratio (M:F)	Age range (years)	Country	Group matching technique	Language exposure	Type of language assessment	Language assessment	Assessed dimensions of language	Assessed languages
Beauchamp et al. (2020)	39	n/s	6;0–9;0	Canada	Not matched	SIM	Direct, indirect	PPVT-IV, ÉVIP-II, CELF-5, CELF-CF, SCQ, M-BLUE	Semantics	L1, L2
Gonzalez-Barrero and Nadig (2017)	52	44:8	5;0–10;0	Canada	Age, NVIQ	n/s	Direct, indirect^a^	PPVT, CELF-IV, CELF-CF, SCQ	Semantics	L1
Gonzalez-Barrero and Nadig (2019b)	40	32:8	6;0–9;0	Canada	Age, NVIQ, dominant language^a^, maternal education	SIM, SEQ	Direct, indirect^b^	PPVT-IV, ÉVIP, TVIP, CELF-IV, CELF-CF, SCQ	Syntax, semantics	DL
Gonzalez-Barrero and Nadig (2019a)	26	22:4	4;9–10;8	Canada	Age, NVIQ, dominant language, maternal education	n/s	Direct	PPVT-IV, ÉVIP, TVIP, CELF-IV, CELF-CF, SCQ	Morphology, semantics	DL, NDL
Hambly and Fombonne (2012)	75	n/s	3;0–6;6	Canada	n/s	SIM, SEQ	Indirect^b^	VABS-II, MCDI, ADI-R, LEI	Semantics, pragmatics	DL, NDL, CV
Hoang et al. (2018)	20	15:5	M: 8;1	Canada	Age^a^, NVIQ^a^, autism symptomatology^a^, vocabulary ability^a^, maternal education^a^	n/s	Direct, indirect^b^	CELF, CELF-CF, EVIP, SCQ	Syntax, semantics, pragmatics	DL
Li et al. (2017)	67	53:14	M: 8;3–9;2	Japan, Canada, United States	Age, Raven Colored Progressive Matrices scores	SIM	Direct, indirect^b^	PPVT-IV, PVT-R, CCC-2	Semantics	L1, L2
Meir and Novogrodsky (2019)	85	49:36	4;0–9;0	Israel	Age, NVIQ, heritage language	SIM, SEQ	Direct	Pronoun elicitation task, LITMUS SRep-30, ADOS	Morphology, syntax	SL
Meir and Novogrodsky (2020)	86	49:37	4;6–9;2	Israel	NVIQ, SES	SIM, SEQ	Direct	LITMUS SRep-30, LITMUS CLT, FWD, BWD, ADOS	Morphology, syntax, semantics	DL, SL
Meir and Novogrodsky (2021)	92	56:36	4;6–9;2	Israel	n/s	SIM, SEQ	Direct	LITMUS SRep-30, LITMUS CLT, ADOS-2	Morphology, syntax	DL, SL
Ohashi et al. (2012)	60	49:11	2;0–4;4	Canada	Age, NVIQ	SIM	Direct, indirect^b^	PLS-4, ADOS, VABS-II, ADI-R	Semantics	L1
Peristeri et al. (2020)	80	80:0	7;3–12;0	Greece	Age	SIM	Direct, indirect^b^	Picture naming test, Sentence repetition task, ENNI	Semantics, syntax	SL
Petersen et al. (2012)	28	26:2	3;7–6;1	Canada	Age	SIM	Direct, indirect^b^	PPVT, PLS-3, PCDI, CDI, CCDI	Semantics	SL, TV, CV
Reetzke et al. (2015)	54	43:11	3;9–8;2	China	n/s	SIM, SEQ	Indirect^b^	CCC-2, SRS, ALEQ, SCQ, LEI	Phonology, morphology, syntax, semantics, pragmatics	DL
Sen and Geetha (2011)	15	8:7	4;0–10;0	India	Language age, SES	n/s	Direct	LPI Hindi, ELTIC	Semantics, morphology, syntax	L1, L2
Sendhilnathan and Chengappa (2020a)	40	29:11	4;0–6;11	Singapore	Age	n/s	Direct	AEPS, Mean length of utterance	Morphology, semantics	L1 or L2
Sendhilnathan and Chengappa (2020b)	40	29:11	4;0–6;11	Singapore	Age	n/s	Direct	AEPS	Pragmatics	L1 or L2
Siyambalapitiya et al. (2022)	120	98:22	M: 3;7	Australia	Chronological age, nonverbal developmental quotient, time between assessments^a^, gender^a^, previous language intervention^a^, childcare attendance prior study entry^a^, adults in household^a^, children in household^a^, number of younger/older siblings^a^, child medication use^a^	n/s	Direct, indirect^b^	SCQ, VABS-II, MSEL	Receptive/ expressive language (n/s)	SL
Valicenti-McDermott et al. (2013)	80	n/a	M: 2;2	United States	n/s	n/s	Direct, indirect^b^	RITLS, clinical observation, VABS	Semantics	n/a^c^
Valicenti-McDermott et al. (2019)	462	369:93	1;0–6;0	United States	n/s	n/s	Direct, indirect^b^	Clinical observation, VABS, parent survey	n/s	n/a^c^
Vanegas (2019)	31	24:7	3;0–12;0	United States	n/s	n/s	Direct, indirect^b^	ROWPVT, EOWPVT, PPVT, EVT, VABS	Phonology, semantics	SL
Zhou et al. (2019)	37	21:16	Baseline: 1;0–2;2	United States	Age, NVIQ	n/s	Direct, indirect^b^	MSEL, VABS-II, MCDI	Semantics	SL

*Note.* SIM: simultaneous; L1: first acquired language; L2: second acquired language; NVIQ: nonverbal IQ; n/s: not specified; SEQ: sequential; DL: dominant language; NDL: non-dominant Language; CV: conceptual vocabulary; SL: societal language; SES: socioeconomic status; TV: total vocabulary scores.

Language assessment: PPVT-IV: Peabody Picture Vocabulary Test, 4th edition; ÉVIP-II: Évaluation de vocabulaire en image Peabody, 2nd edition; CELF-5: Clinical Evaluation of Language Fundamentals, 5th edition; CELF-CF: Clinical Evaluation of Language Fundamental-Version Canadienne Française; SCQ: Social Communication Questionnaire; M-BLUE: Montréal Bilingual Language Use and Exposure; PPVT: Peabody Picture Vocabulary Test; CELF-IV: Clinical Evaluation of Language Fundamentals, 4th edition; ÉVIP: Évaluation de vocabulaire en image Peabody; TVIP: Test de Vocabulario en Imagenes Peabody; VABS-II: Vineland Adaptive Behavior Scales, 2nd edition; MCDI: MacArthur-Bates Communicative Development Inventory; ADI-R: Autism Diagnostic Interview-Revised; LEI: Language Environment Interview; PVT-R: Picture Vocabulary Test—Revised, Japanese Version; CCC-2: Children’s Communication Checklist–2; LITMUS SRep-30: LITMUS Sentence-Repetition task; ADOS: Autism Diagnostic Observation Schedule; LITMUS CLT: LITMUS Cross-linguistic lexical task; FWD: Hebrew Forward Digit Span of the Wechsler Intelligence Scale for Children; BWD: Hebrew Backward Digit Span of the Wechsler Intelligence Scale for Children; ADOS-2: Autism Diagnostic Observation Schedule, 2nd edition; PLS-4: Preschool Language Scale, 4th edition; ENNI: Edmonton Narrative Norms Instrument; PLS-3: The Preschool Language Scale; PCDI: Putonghua Communicative Development Inventories; CDI: Communicative Development Inventories; CCDI: Chinese Communicative Development Inventories; SRS: Social Responsiveness Scale; ALEQ: Alberta Language Environment Questionnaire; LPI Hindi: Linguistic Profile Test Hindi; ELTIC English Language Testing for Indian Children; AEPS: Assessment, Evaluation, and Programming System for Infants and Children; MSEL: Mullen Scales of Early Learning; RITLS: Rossetti Infant-Toddler Language Scale; VABS: Vineland Adaptive Behavior Scales; ROWPVT: Receptive One-Word Picture Vocabulary Test; EOWPVT: Expressive One-Word Picture Vocabulary Test; EVT: Expressive Vocabulary Test.

a Indicates that this group matching technique could not be applied to all participants.

b Indicates that the indirect measure was a parent report.

c Only (nonverbal) communicative measures were reported.

A total of 11 of the 22 publications had overlapping participant samples, which limited the synthesis of the findings. While the composition of the subgroups was different for each of the studies, Hoang et al. (2018) and the publications by Gonzalez-Barrero and Nadig (2017, 2019a, 2019b) drew their participants from the same larger study. The two publications by Sendhilnathan and Chengappa (2020a, 2020b) were based on the same study and included the same participants. Based on the description of participant recruitment, there was also a significant overlap in participants in the publications by Valicenti-McDermott et al. (2013, 2019) and Meir and Novogrodsky (2019, 2020, 2021).

### Language assessment and the representation of the five dimensions of language

The 22 studies included in the current review assessed different dimensions of languages and different skills within these dimensions. Semantic-related skills, such as vocabulary scores, were the most frequently reported dimension of language (*n* = 18). A total of 62% of the studies (*n* = 13) reported scores for expressive or receptive vocabulary (e.g. Vanegas, 2019; Zhou et al., 2019). Eight studies reported assessments of syntactic skills, such as sentence repetition (e.g. Hoang et al., 2018). Seven studies assessed morphological skills (e.g. Gonzalez-Barrero & Nadig, 2019a). Four studies assessed pragmatic-related skills (e.g. Hambly & Fombonne, 2012; Reetzke et al., 2015), and only two studies (Reetzke et al., 2015; Vanegas, 2019) reported results related to participants’ phonologic skills.

Thirteen studies used both direct and indirect measures to assess participants’ language and communication skills (e.g. Ohashi et al., 2012; Peristeri et al., 2020). Direct assessments included direct observations or assessments, while indirect language assessments included information reported through a parent questionnaire. Seven studies used only direct assessment tools (e.g. Meir & Novogrodsky, 2019, 2020, 2021), and two studies only used indirect assessments (Hambly & Fombonne, 2012; Reetzke et al., 2015).

Different editions of the Peabody Picture Vocabulary Test (PPVT; Dunn & Dunn, 2007) were the most frequently administered direct assessment tool (*n* = 8), followed by the Clinical Evaluation of Language Fundamentals (CELF; Wiig et al., 2013; *n* = 5). The most commonly used indirect assessments were the Social Communication Questionnaire (SCQ; Rutter et al., 2003; *n* = 7) and the VABS (Sparrow et al., 2005; *n* = 7). Other examples of indirect assessments were the MCDI (Fenson et al., 2007) and the Children’s Communication Checklist (CCC; Bishop, 2006), which were each used by two of the included studies.

Twelve studies only reported scores for one language for multilingual participants, generally for the first language (e.g. Ohashi et al., 2012), societal majority language (e.g. Zhou et al., 2019), or dominant language (e.g. Gonzalez-Barrero & Nadig, 2019b). Only seven studies reported scores for both languages for multilingual participants (see Table 1). In addition to reporting scores for participants’ dominant and non-dominant language, Hambly and Fombonne (2012) also reported participants’ conceptual vocabulary scores. Petersen et al. (2012) only reported scores for the societal language (English) and not for the participants’ home language but also reported total and conceptual vocabulary scores. Valicenti-McDermott et al. (2013, 2019) only reported results for communicative measures.

### Comparison of the language and communication skills of multilingual children on the autism spectrum and their peers

Eleven studies compared the scores of multilingual autistic children only with their monolingual autistic peers (e.g. Ohashi et al., 2012; Petersen et al., 2012; Reetzke et al., 2015). Nine studies (e.g. Beauchamp et al., 2020; Meir & Novogrodsky, 2021) compared four different groups of participants: monolingual autistic children, multilingual autistic children, monolingual nonautistic children, and multilingual nonautistic children. The publication by Hambly and Fombonne (2012) was the only study that reported scores separately for simultaneous and sequential bilinguals in comparison with monolingual autistic children. The study by Sen and Geetha (2011) was unique because they separated the monolingual participants into two groups according to their language (Hindi, English). The most common group matching criteria were age and nonverbal IQ.

We coded and analyzed the language and communication skills reported in the 22 studies. Out of the core areas of linguistics (Skandera & Burleigh, 2016), the reviewed publications most frequently assessed semantics (*n* = 18) and syntax (*n* = 8). The most frequently evaluated skill was vocabulary scores (*n* = 13). For example, Hambly and Fombonne (2012) found that bilingual children generally presented with significantly smaller vocabularies in their second language and often had not achieved phrase-level speech in their second language.

Phonetics and phonology-related skills were only reported indirectly by Vanegas (2019) and Reetzke et al. (2015). Reetzke et al. (2015) reported scores for the speech subcategory of the CCC-2 (Bishop, 2006) but did not separately analyze these scores. Vanegas (2019) found no effect of bilingualism on phonemic awareness in children on the autism spectrum.

Seven studies specifically assessed morphological skills. For example, Meir and Novogrodsky (2019) assessed pronoun use as one measure of morphosyntax. Gonzalez-Barrero and Nadig (2019a) found no significant differences between monolingual and bilingual children on the autism spectrum regarding morphological skills.

Sentence repetition was frequently used (*n* = 7) to assess syntactic abilities (e.g. Peristeri et al., 2020). Pragmatic measures were assessed by only four studies (e.g. Hoang et al., 2018). Regarding nonverbal communication, Valicenti-McDermott et al. (2013) analyzed communicative measures, including pointing, gesturing, and making eye contact, and found a bilingual advantage in some of the measures. Zhou et al. (2019) found that bilingual children started with lower gesture use but made greater gains over time than their monolingual peers.

## Discussion

The current review aimed to answer two main research questions: (a) What dimensions of language have been included in studies of multilingualism in autistic children and how they have been measured, and (b) How the language and communication skills of multilingual autistic children compared with the skills of multilingual nonautistic children and monolingual autistic children.

### Dimensions of language and language measurement

The 22 publications included in the current review addressed the five dimensions of language (phonology, morphology, semantics, syntax, and pragmatics) to varying degrees. Findings related to semantics (e.g. vocabulary scores) were most frequently reported. The most underreported language dimensions were phonology and pragmatic-related skills, including nonverbal and preverbal communication skills. As pragmatic-related skills are frequently an area of difficulty for children on the autism spectrum, this gap in research is particularly concerning.

Most studies (*n* = 13) used direct and indirect measures to assess language and communication skills. The combination of direct and indirect measures provides a more accurate reflection of children’s language and communication skills, as direct assessments generally only capture one moment, frequently in clinical environments. In contrast, parent assessments can provide a more longitudinal reflection of natural settings.

The 22 studies synthesized in this review employed a variety of assessment tools, for example, the PPVT (Dunn & Dunn, 2007) and the SCQ (Rutter et al., 2003). However, only seven studies reported bilingual participants’ language and communication scores for both languages. In concurrence with other publications (e.g. MacSwan & Rolstad, 2006), Meir and Novogrodsky (2020) argued that inadequate assessment tools could lead to misrepresentation of the language abilities of multilingual children. Meir and Novogrodsky also discussed that had they tested bilingual children in both languages (i.e. their dominant language and the societal language), there might have been a bilingual advantage. This hypothesis aligns with the criticism of the inaccuracy of single-language measures for multilingual populations (e.g. Core et al., 2013). Out of the included studies, only Petersen et al. (2012) reported total and conceptual vocabulary scores, and Hambly and Fombonne (2012) reported conceptual vocabulary scores. Future studies should include total vocabulary scores to reflect the most accurate multilingual language skills assessment method.

### Impact of multilingualism on language and communication skills

The studies analyzed in this review did not provide enough evidence to allow conclusions about the impact of bilingualism on the phonetic and phonological skills of autistic children. Regarding morphology, the reviewed research has identified multiple differences between morphologic skills of autistic children and their nonautistic peers (Gerenser & Lopez, 2017). Meir and Novogrodsky (2019) found that nonautistic children generally outperformed their autistic peers on morphological tasks. No significant differences, however, were found between monolingual and bilingual autistic children (Gonzalez-Barrero & Nadig, 2019a; Meir & Novogrodsky, 2019).

Findings on receptive and expressive vocabulary skills of bilingual autistic children were contradictory. Four studies concluded that there were no significant differences in vocabulary scores between monolingual and bilingual children in both the autistic and nonautistic participant groups (Gonzalez-Barrero & Nadig, 2017; Ohashi et al., 2012; Petersen et al., 2012 & Vanegas, 2019). Other studies reported that bilingual children scored lower on both receptive (Gonzalez-Barrero & Nadig, 2019a; Hoang et al., 2018; Meir & Novogrodsky, 2020) and expressive (Peristeri et al., 2020) vocabulary scores compared with their monolingual peers. However, this may be due to the use of single-language comparisons (Core et al., 2013) instead of total vocabulary scores. In total, only three studies (Beauchamp et al., 2020; Hambly & Fombonne, 2012; Meir & Novogrodsky, 2020) reported vocabulary scores for both languages of bilingual participants. Again, future research should include assessment methods in *both* languages of bilingual participants in order to accurately measure vocabulary development and skills. Doing so will allow for more valid comparisons across groups.

Echolalia, the immediate or delayed reproduction of utterances (Grossi et al., 2013), is a common behavioral characteristic of autism (APA, 2013). Echolalia was not addressed by any study included in this review, which is of particular importance considering that sentence repetition was frequently used to assess syntactic skills (e.g. Peristeri et al., 2020). Other syntactic skills where bilingual effects have been observed, such as syntactic parsing (e.g. Dussias & Sagarra, 2007), have not yet been assessed in bilingual children on the autism spectrum.

Social communication difficulties are a main diagnostic criterion for autism (APA, 2013); however, only a few studies included pragmatic and nonverbal skills. Joint attention, an early developmental milestone frequently delayed in children on the autism spectrum (APA, 2013), was one of the few preverbal skills assessed by the studies included in this review. Three studies (Hambly & Fombonne, 2012; Ohashi et al., 2012; Peristeri et al., 2020) assessed joint attention through the Autism Diagnostic Interview-Revised (ADI-R; Le Couteur et al., 2003). Hambly and Fombonne (2012) concluded there was no negative effect of bilingualism on early social communication skills such as joint attention. Other studies reported a bilingual advantage in some communicative measures like gesture use (Zhou et al., 2019) and pointing (Valicenti-McDermott et al., 2013) for bilingual autistic children. Concurringly, a longitudinal single-case study by Seung et al. (2006) reported an increase in nonverbal communication skills, including eye contact, for the bilingual participant.

Both differences and similarities have been reported for the language and communication skills of multilingual children on the autism spectrum in relation to their peers’ skills. Positive effects of bilingualism, similar to the effects that have been reported for nonautistic populations, were indicated for autistic children on some measures, such as verbal fluency (Gonzalez-Barrero & Nadig, 2017). Multilingual autistic children, however, also shared many characteristics with their monolingual autistic peers, including deficits in morphological (e.g. Meir & Novogrodsky, 2019) and pragmatic skills (e.g. Hoang et al., 2018). To date, studies have not assessed whether common bilingual phenomena such as code-switching (Paradis et al., 2021) and typical autism characteristics such as echolalia are equally common in bilingual children on the autism spectrum as they are in the language of their peers.

In summary, included publications varied significantly regarding terminology, eligibility criteria, group matching, and represented languages. Many studies excluded children with a co-occurring intellectual disability, children who were exposed to more than two languages, and participants with complex communication needs. The participant data indicate that the few publications on multilingualism in children on the autism spectrum do not encompass the whole autism spectrum. This limits the generalizability of the findings that were synthesized in the current review.

### Additional implications, recommendations for future research, and limitations

There are indications of positive effects of bilingualism, for example, in verbal fluency (Gonzalez-Barrero & Nadig, 2017). Seemingly negative effects of bilingualism, such as lower scores on syntactic abilities, generally became insignificant when analyses controlled for vocabulary scores (e.g. Meir & Novogrodsky, 2020). This finding aligns with previous studies on multilingual children (e.g. Komeili & Marshall, 2013; Meir, 2017). In summary, the available evidence does not support the hypothesis that multilingualism poses unique barriers to the language and communication development of children on the autism spectrum. This is especially important as studies have shown that speech-language pathologists, teachers, and other service providers often advise parents of children on the autism spectrum not to provide multilingual environments (Fernandez y Garcia et al., 2012).

Many factors influence the language and communication skills of children on the autism spectrum: Both external and internal factors can contribute to a delay in language development (Komeili & Marshall, 2013). Along with influential factors such as time and amount of exposure (Luk & Bialystok, 2013), changes in the language environment are another possible contributor. Changes in language exposure over children’s lifetime were only specifically addressed by Hambly and Fombonne (2012). Future studies should include more information regarding exposure and other contextual factors. As a number of studies have investigated cognitive skills, for example, executive functioning (e.g. Li et al., 2017), we want to highlight that it is important for future research to conduct a systematic review of cognitive skills of multilingual autistic children to extend the findings regarding language and communication skills.

No review is without limitations. A central limitation of the current review is that only group comparisons have been included. Single-case and qualitative studies, such as interviews, have not been included in the current review. In addition, all studies that met the eligibility criteria of the current review were included, regardless of study quality.

## Conclusion

This systematic review synthesized the findings of 22 peer-reviewed articles. Some dimensions of language, such as syntax and semantics, are represented well in the available research, while other areas, such as phonology and pragmatics, are severely understudied.

The findings of this review provide no evidence that being exposed to more than one language has any negative effects on the language and communication skills of autistic children. Multilingual autistic children often have common autism characteristics affecting their communication in a manner similar to their monolingual autistic peers. However, preliminary findings also indicate that bilingual autistic children may share some advantages of bilingualism with their bilingual nonautistic peers.
